# Synthesis and structure of pyridinium tri­chlorido­(pyridine-κ*N*)zincate(II)

**DOI:** 10.1107/S2056989026007061

**Published:** 2026-07-16

**Authors:** Guzal Rakhmatova, Iskandar Allaberdiyev, Kyzlarkhan Siddikova, Jasur Makhmayorov, Laylo Parmonova, Anvar Xudoyqulov

**Affiliations:** a Karshi State Technical University, 225 Mustaqillik Avenue, Karshi City, Kashkadarya region, Uzbekistan; b UzGTL, Guzar district, Kashkadarya region, Uzbekistan; c University of Economics and Pedagogy, 13 I. Karimov Avenue, Karshi, 180100, Uzbekistan; University of Aberdeen, United Kingdom

**Keywords:** crystal structure, zinc(II) com­plex, pyridine, chlorido­zincate, Hirshfeld surface analysis, π–π stacking inter­actions

## Abstract

In the title salt, (C_5_H_6_N)[ZnCl_3_(C_5_H_5_N)], the Zn^2+^ atom is four-coordinated by one pyridine N atom and three chloride ligands, forming a slightly distorted tetra­hedral ZnNCl_3_ environment. In the crystal, the cation and anion are linked by bifurcated N—H⋯(Cl,Cl) and C—H⋯Cl hy­dro­gen bonds, generating a supra­molecular assembly that is further consolidated by π–π stacking inter­actions between aromatic rings. Hirshfeld surface analysis shows that H⋯Cl/Cl⋯H contacts are the dominant contribution to the crystal packing, accounting for 44.1% of the total surface contacts, followed by H⋯H (29.9%) and H⋯C/C⋯H (15.0%) inter­actions.

## Abstract 2

In the title salt, (C_5_H_6_N)[ZnCl_3_(C_5_H_5_N)], pyridine acts as a protonated counter-ion and a ligand in the com­plex anion.

## 1. Chemical context

A large number of com­pounds based on transition-metal com­plexes with pyridine and its derivatives have been reported in the literature (Khan, 2021 ▸; Elsayed *et al.*, 2024 ▸; Zou *et al.*, 2021 ▸). Pyridine is one of the simplest heterocyclic com­pounds, structurally resembling benzene, in which a methine group is replaced by an N atom. It is precisely this electronegative N atom that fundamentally distinguishes pyridine from benzene, endowing it with distinct chemical properties. The pyridine scaffold is one of the most important structural elements found in numerous approved pharmaceutical drugs on the market (Khan, 2021 ▸; Temple *et al.*, 1992 ▸; El-Naggar *et al.*, 2021 ▸).
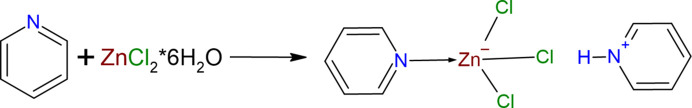


Unlike many other transition metals, the Zn^2+^ ion has a 3*d*^10^ electronic configuration, displays no redox activity and is generally far less toxic to humans, which makes it a promising basis for the development of metal-containing drugs (Porchia *et al.*, 2020 ▸). Zinc typically forms com­plexes with coordination numbers of 4 or 6, with tetra­hedral coordination being the most characteristic geometry for this ion (Kokunov *et al.*, 2009 ▸).

In recent years, inter­est in the synthesis of zinc-based com­plexes has grown considerably owing to their favourable biological properties, with pyridine and its derivatives being among the most effective ligands for this purpose. Such com­plexes have been synthesized and characterized in several studies (Modec, 2018 ▸; Sun *et al.*, 2022 ▸). It has also been shown that some pyridine-based com­plexes exhibit higher biological activity than the corresponding free ligand. This is attributed to a synergistic effect between the metal centre and the biologically active ligand, as well as to an increase in the lipophilicity of the com­plex and, consequently, its ability to cross cell membranes. Xun-Zhong *et al.* (2020 ▸) synthesized zinc com­plexes with two pyridine-derived ligands that displayed higher anti­tumour and anti­bacterial activity than the free ligand. Likewise, a zinc com­plex based on an imino­pyridine ligand was found to exhibit higher anti­bacterial activity against planktonic cells of *Staphylococcus aureus* (Gram-positive) and *Escherichia coli* (Gram-negative) strains than the corresponding copper com­plex (de la Mata Moratilla *et al.*, 2024 ▸).

As part of our studies in this area, we now describe the synthesis and structure of the title salt, (C_5_H_6_N)[ZnCl_3_(C_5_H_5_N)], (**I**).

## 2. Structural commentary

**Figure 1:**
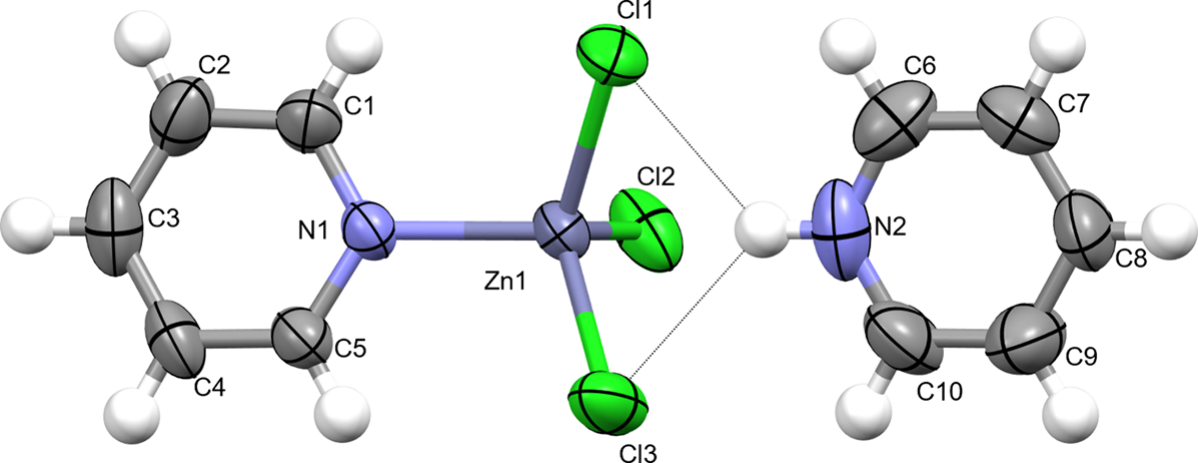
The mol­ecular structure of (**I**), with displacement ellipsoids for non-H atoms drawn at the 50% probability level. Hydrogen bonds are shown as dashed lines.

Compound (**I**) crystallizes in the monoclinic space group *P*2_1_/*n* and the asymmetric unit contains one [C_5_H_6_N]^+^ pyridinium cation and one [ZnCl_3_(C_5_H_5_N)]^−^ anion. The Zn^2+^ ion is coordinated by three chloride ions and one pyridine N atom, forming a slightly distorted tetra­hedral coordination environment (Fig. 1 ▸). The Zn—N bond length is 2.049 (2) Å, whereas the Zn—Cl distances lie in the typical range observed for tetra­hedral chlorido­zinc(II) com­plexes (Cotton *et al.*, 1985 ▸; Albrecht *et al.*, 2003 ▸), namely, 2.2295 (8)–2.2551 (9) Å (Table 1 ▸). The bond angles around the metal centre vary from 106.42 (7) to 114.48 (4)°, deviating from the ideal tetra­hedral value of 109.47°. The largest angle is Cl2—Zn—Cl3 = 114.48 (4)°, whereas the smallest is Cl2—Zn—N1 = 106.42 (7)°. The extent of distortion is small, as confirmed by the τ_4_ parameter (Addison *et al.*, 1984 ▸) of 0.946, which is close to unity for an ideal tetra­hedron. As expected, the pyridine ring of the coordinated ligand is essentially planar, with an r.m.s. deviation of 0.006 Å. In the counter-ion, protonation of the N atom gives rise to a pyridinium cation, which com­pensates the negative charge of the com­plex anion.

## 3. Supra­molecular features

**Figure 2:**
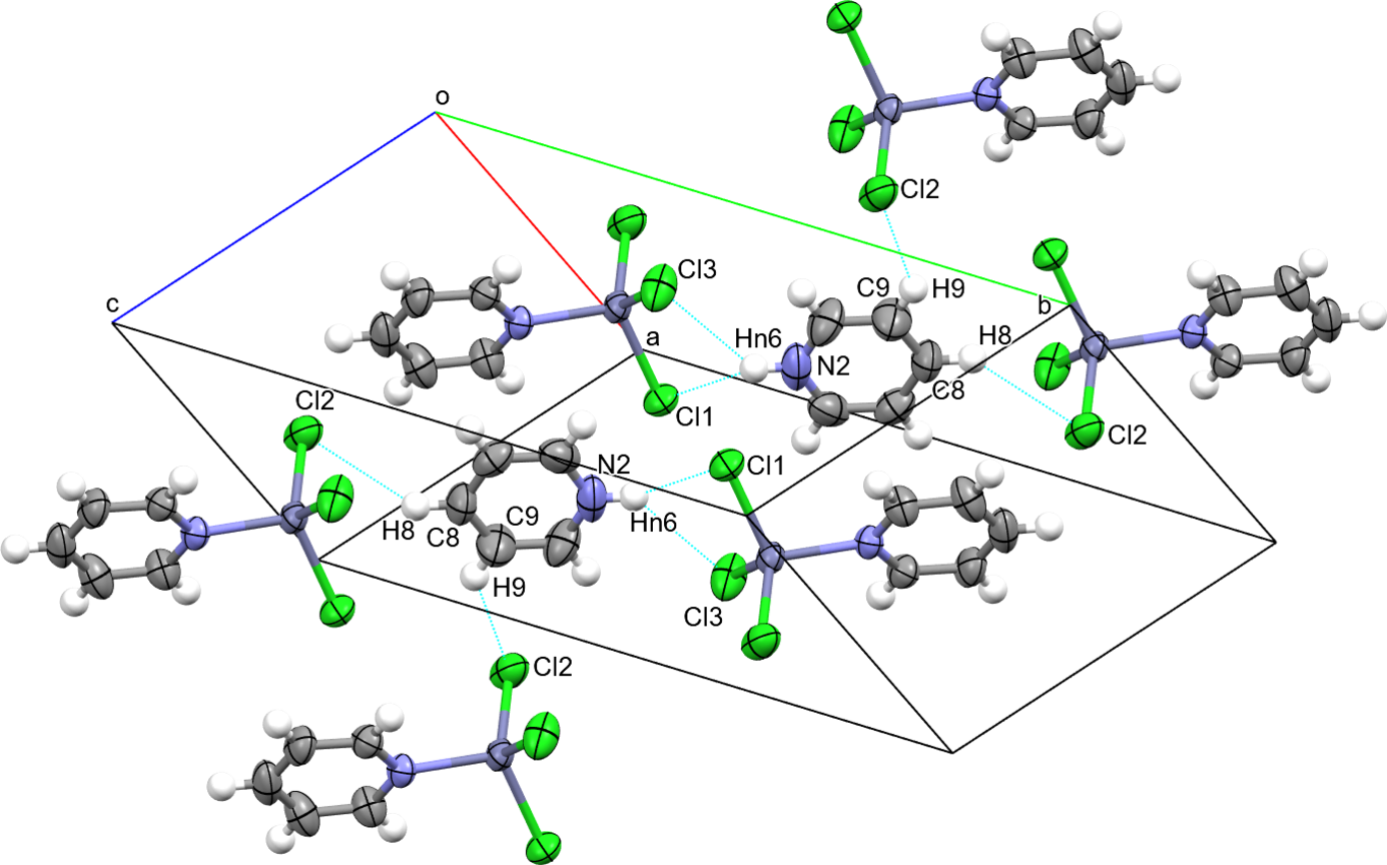
Supra­molecular structure of (**I**), showing N—H⋯Cl hy­dro­gen bonds and non-classical C—H⋯Cl inter­actions forming chains along [120].

The crystal packing of com­pound (**I**) features bifurcated N—H⋯(Cl,Cl) hy­dro­gen bonds, as well as non-classical C—H⋯Cl inter­actions (Fig. 2 ▸). The protonated N2 atom of the pyridinium cation acts as an efficient hy­dro­gen-bond donor and forms a bifurcated inter­action with the chloride ions of the com­plex anion, namely, N2—HN6⋯Cl1 and N2—HN6⋯Cl3 (Table 2 ▸). The H⋯Cl separations are almost the same and the Cl⋯H⋯Cl angle is 84.2 (4)°.

In addition, the packing is consolidated by two C—H⋯Cl contacts. Atoms H8 and H9 of the pyridine ring participate in inter­actions with atom Cl2 of neighbouring com­plex anions (Table 2 ▸ and Fig. 2 ▸). Thus, the C—H⋯Cl inter­actions com­plement the classical N—H⋯Cl contacts and generate an extended supra­molecular network.

**Figure 3:**
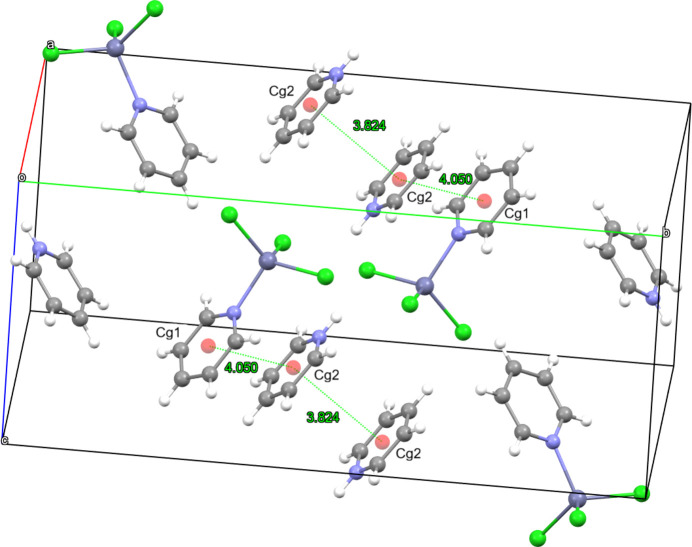
Aromatic π–π stacking inter­actions in the crystal structure of (**I**).

An additional stabilizing factor is provided by weak π–π stacking inter­actions between adjacent aromatic rings (Fig. 3 ▸), with centroid–centroid separations of *Cg*2⋯*Cg*2 = 3.824 (2) Å and *Cg*1⋯*Cg*2 = 4.051 (2) Å, where *Cg*1 denotes the C1–C5/N1 ring and *Cg*2 denotes the C6–C10/N2 ring. These values, together with the moderate inter­planar angles and noticeable lateral displacement of the rings, indicate a slipped π–π stacking arrangement. Although these inter­actions are not strong in the classical sense, they appear to make a significant contribution to the consolidation of the crystal packing.

## 4. Hirshfeld surface and void analysis

**Figure 4:**
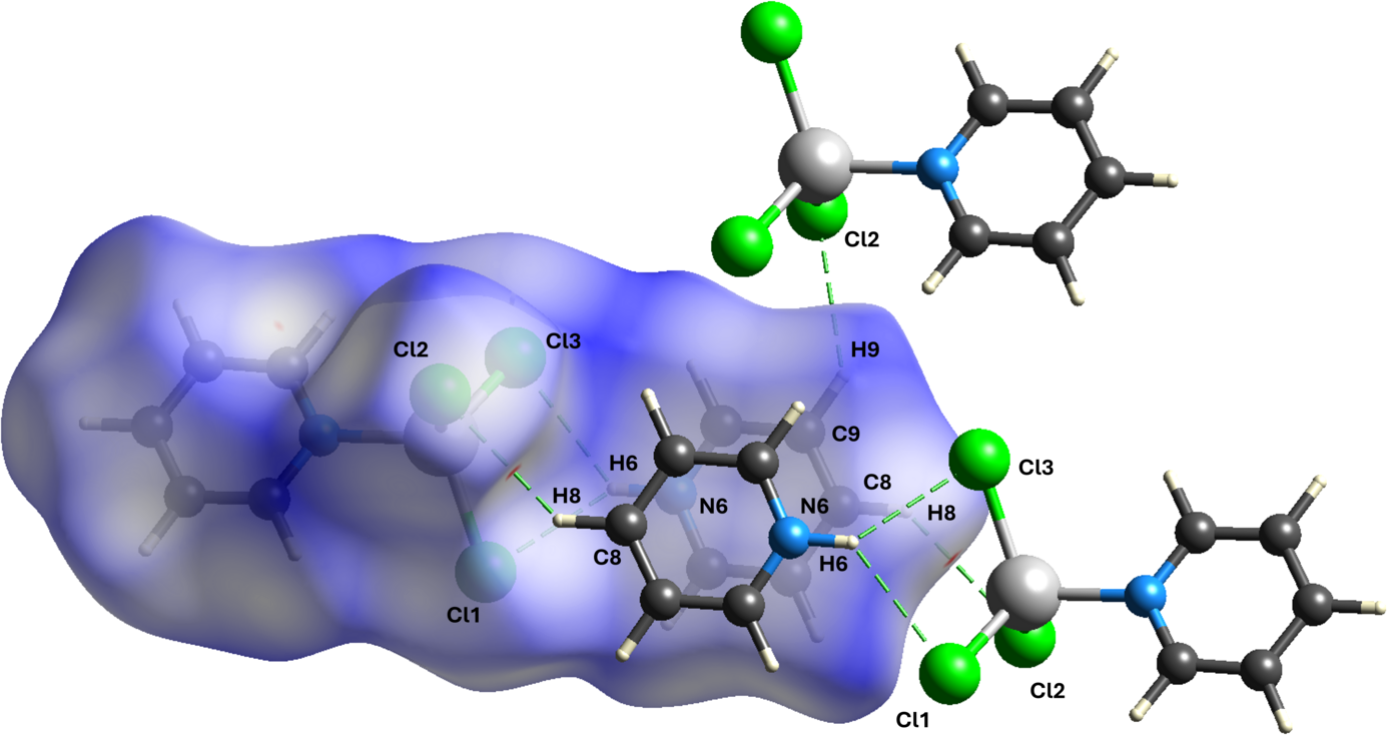
Hirshfeld surface of (**I**) mapped over *d*_norm_, highlighting close inter­molecular contacts as red spots corresponding to regions of strong hy­dro­gen-bonding inter­actions.

The Hirshfeld surface (HS) of (**I**) was calculated using *CrystalExplorer 21.5* (Spackman *et al.*, 2021 ▸). The *d*_norm_ map (Fig. 4 ▸) exhibits characteristic red spots in regions of the shortest inter­molecular contacts, indicating areas where inter­atomic distances are shorter than the sum of the corresponding van der Waals radii. In the present structure, the most prominent contacts are associated with N—H⋯Cl and C—H⋯Cl inter­actions, as well as with close contacts between aromatic fragments. The blue regions on the surface correspond to inter­molecular distances that exceed the van der Waals separations and therefore make a smaller contribution to the direct association of mol­ecular fragments.

**Figure 5:**
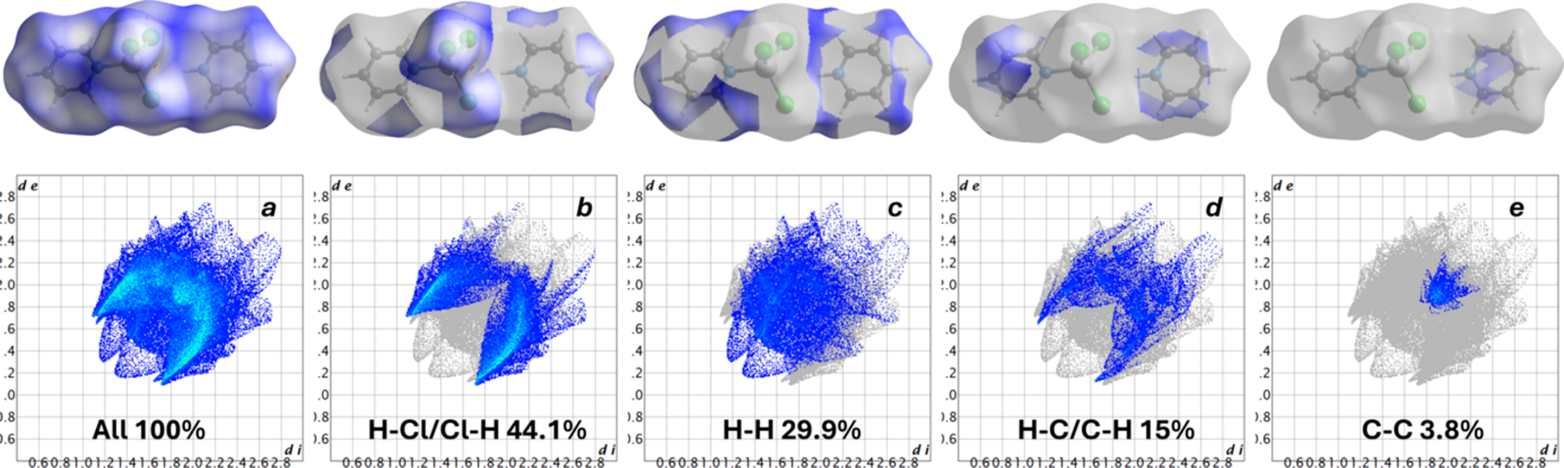
Two-dimensional fingerprint plots for (**I**), showing all inter­actions (*a*) and delineated into selected inter­actions: (*b*) H⋯Cl/Cl⋯H, (*c*) H⋯H, (*d*) H⋯C/C⋯H and (*e*) C⋯C, together with their relative contributions to the Hirshfeld surface.

The total HS area and volume are 313.6 Å^2^ and 338.6 Å^3^, respectively. The two-dimensional fingerprint plot provides a qu­anti­tative overview of all pairwise inter­molecular contacts in the crystal packing and allows the relative importance of the different inter­action types to be assessed (Fig. 5 ▸). The largest contribution to the Hirshfeld surface arises from H⋯Cl/Cl⋯H contacts, accounting for 44.1% [Fig. 5 ▸(*b*)], which is fully consistent with the chloride-rich nature of the com­plex and the dominant role of weak hy­dro­gen-bonding and halogen–acceptor inter­actions in consolidating the packing. Significant contributions are also made by H⋯H contacts [29.9%; Fig. 5 ▸(*c*)], reflecting van der Waals inter­actions between non-polar fragments, and H⋯C/C⋯H contacts [15.0%; Fig. 5 ▸(*d*)], which are generally associated with C—H⋯π inter­actions and close contacts between aromatic rings.

Smaller, but still noticeable, contributions are provided by C⋯C contacts (3.8%), indicating aromatic stacking; N⋯C/C⋯N (2.5%) and N⋯H/H⋯N (2.3%), reflecting the participation of N atoms in additional weak inter­molecular contacts; Cl⋯N/N⋯Cl (1.0%) and Cl⋯C/C⋯Cl (0.7%), corresponding to secondary halogen-containing inter­actions; and Zn⋯H/H⋯Zn (0.5%) and Cl⋯Cl (0.2%), which make only a minor contribution to the overall packing.

**Figure 6:**
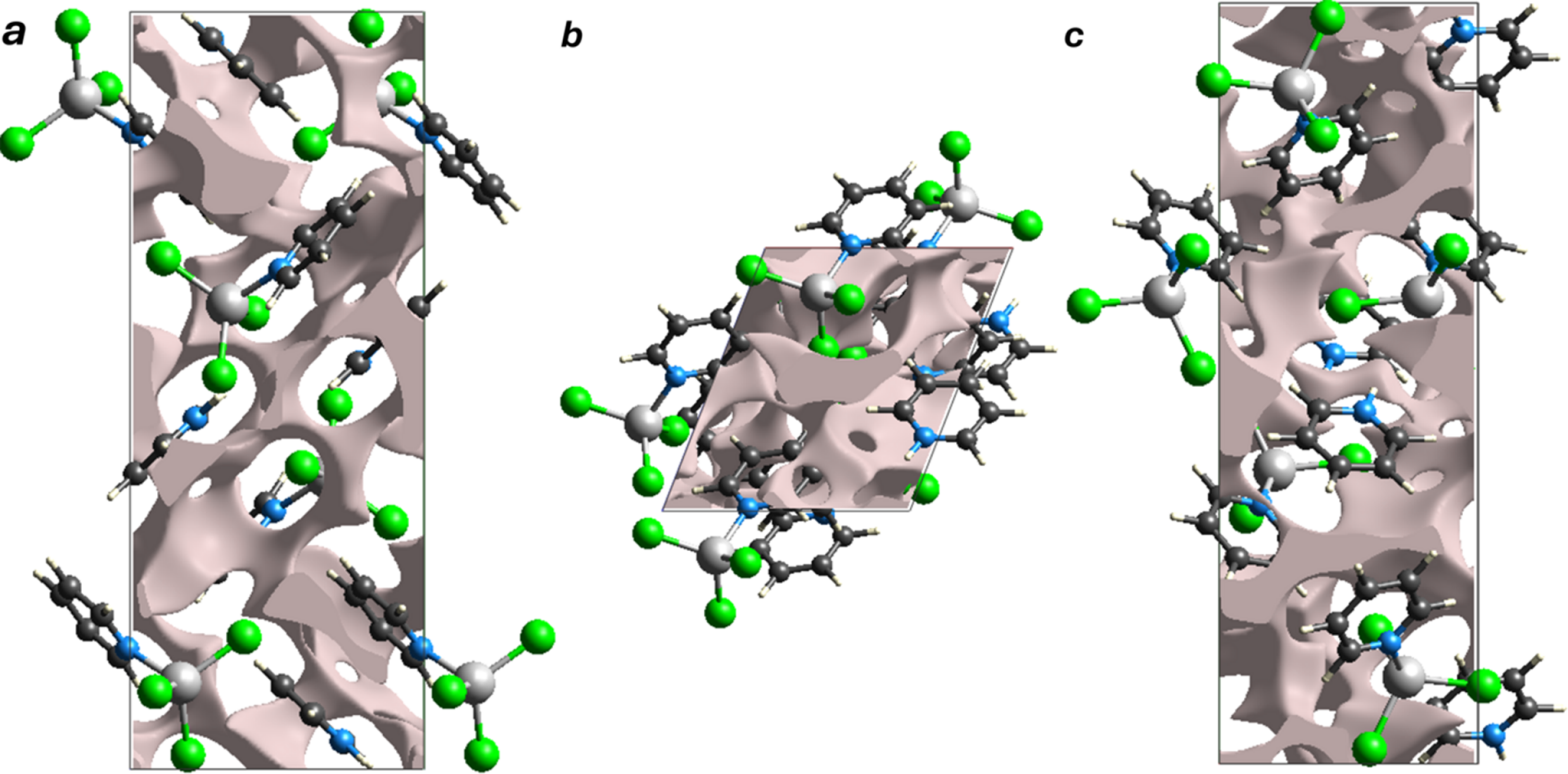
The void surface of (**I**).

Void analysis based on the promolecular electron density shows that the crystal packing is relatively dense and does not contain an extended channel system (Fig. 6 ▸). The void volume is 160.5 Å^3^ and its surface area is 549.6 Å^2^. The identified cavities are localized and do not form a continuous porous network, indicating a predominantly com­pact packing arrangement in the crystal. Visual inspection of the void surface reveals that no significant voids are present between neighbouring cationic and anionic species owing to strong electrostatic attraction and the network of N—H⋯Cl and C—H⋯Cl hy­dro­gen bonds. Instead, the void surface surrounds the ionic assembly as a whole, indicating that the residual free space is distributed around the cation–anion aggregates rather than being localized between oppositely charged fragments. The presence of such a limited void is consistent with the observed set of weak inter­molecular inter­actions, which link the com­ponents into a stable but non-porous supra­molecular motif. For density functional theory (DFT) calculations, see supporting information.

## 5. Database survey

A search of the Cambridge Structural Database (CSD, Version 2026.2.0; Groom *et al.*, 2016 ▸) identified five structurally related com­plexes. In all of these entries, pyridine or pyridine-derived ligands are present both as coordinated donors and as hy­dro­gen-bonded species, and chloride ions are also structural com­ponents. The closest structural analogue is the zinc com­plex reported by Jin *et al.* (2005 ▸), in which 2-amino-5-methyl­pyridine acts as the ligand (CSD refcode FOBKOX). A related ruthenium com­plex containing the same ligand was also found (Webb *et al.*, 2013 ▸), in which two pyridine mol­ecules are coordinated to the central metal atom together with four chloride ligands, while an additional pyridine mol­ecule is involved in hy­dro­gen-bonding inter­actions (DIPYAE).

## 6. Synthesis and crystallization

The following solutions were prepared: (*a*) an ethano­lic solution of a ZnCl_2_·6H_2_O (1.0 mmol) and (*b*) an ethano­lic solution of pyridine (2.0 mmol). Solution (*a*) was added to solution (*b*), and the resulting mixture was stirred at room tem­per­a­ture for 12 h using magnetic stirring. A crystalline precipitate was formed, filtered off, washed several times with ethanol and dried in air. Since the obtained material was readily soluble in di­methyl­formamide (DMF), it was recrystallized from this solvent, yielding well-formed yellow single crystals suitable for X-ray diffraction analysis and further physicochemical investigations.

## 7. Refinement

Crystal data, data collection and structure refinement details are summarized in Table 3 ▸. H atoms bonded to C and N atoms were placed in calculated positions and refined using a riding model, with *U*_iso_(H) = 1.2*U*_eq_(C,N).

## Supplementary Material

Crystal structure: contains datablock(s) I. DOI: 10.1107/S2056989026007061/hb8233sup1.cif

CCDC reference: 2571358

Additional supporting information:  crystallographic information; 3D view; checkCIF report

## Figures and Tables

**Table 1:** Selected geometric parameters (Å, °) *Cg*1 denotes the C1–C5/N1 ring and *Cg*2 denotes the C6–C10/N2 ring

Zn1—Cl1	2.2551 (7)	Zn1—Cl3	2.2451 (9)
Zn1—Cl2	2.2295 (8)	Zn1—N1	2.049 (2)
			
*Cg*1⋯*Cg*2^i^	4.051 (2)	*Cg*2⋯*Cg*2^ii^	3.824 (2)
			
Cl2—Zn1—Cl1	112.16 (3)	N1—Zn1—Cl1	107.33 (6)
Cl3—Zn1—Cl1	109.25 (3)	N1—Zn1—Cl2	106.42 (7)
Cl3—Zn1—Cl2	114.48 (4)	N1—Zn1—Cl3	106.76 (7)

Symmetry codes: (i) 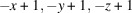; (ii) 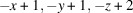.

**Table 2:** Hydrogen-bond geometry (Å, °)

*D*—H⋯*A*	*D*—H	H⋯*A*	*D*⋯*A*	*D*—H⋯*A*
N2—HN6⋯Cl1	0.86 (1)	2.74 (1)	3.389 (3)	133 (1)
N2—HN6⋯Cl3	0.86 (1)	2.74 (1)	3.388 (5)	134 (1)
C8—H8⋯Cl2^ii^	0.93 (1)	2.92 (1)	3.681 (5)	140 (1)
C9—H9⋯Cl2^iii^	0.93 (1)	2.93 (1)	3.669 (5)	137 (1)

Symmetry codes: (ii) 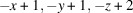; (iii) 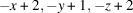.

**Table 3:** Experimental details

Crystal data	
Chemical formula	(C_5_H_6_N)[ZnCl_3_(C_5_H_5_N)]
*M*_r_	330.97
Crystal system, space group	Monoclinic, *P*2_1_/*n*
Temperature (K)	293
*a*, *b*, *c* (Å)	7.80795 (16), 21.3634 (4), 8.90581 (18)
β (°)	111.418 (2)
*V* (Å^3^)	1382.94 (5)
*Z*	4
Radiation type	Cu *K*α
μ (mm^−1^)	7.60
Crystal size (mm)	0.62 × 0.45 × 0.20

Data collection	
Diffractometer	Rigaku XtaLAB Synergy diffrac­tometer with a HyPix3000 detector
Absorption correction	Gaussian (*CrysAlis PRO*; Rigaku OD, 2023 ▸)
*T*_min_, *T*_max_	0.326, 1.000
No. of measured, independent and observed [*I* ≥ 2σ(*I*)] reflections	7075, 2517, 2205
*R*_int_	0.041
(sin θ/λ)_max_ (Å^−1^)	0.602

Refinement	
*R*[*F*^2^ > 2σ(*F*^2^)], *wR*(*F*^2^), *S*	0.044, 0.126, 1.02
No. of reflections	2517
No. of parameters	146
H-atom treatment	H-atom parameters constrained
Δρ_max_, Δρ_min_ (e Å^−3^)	0.52, −0.73

Computer programs: *CrysAlis PRO* (Rigaku OD, 2023 ▸), *SHELXT* (Sheldrick, 2015 ▸), *olex2.refine* (Bourhis *et al.*, 2015 ▸) and *OLEX2* (Dolomanov *et al.*, 2009 ▸).

## supplementary crystallographic information

### Pyridinium trichlorido(pyridine-κN)zincate(II) Crystal data

**Table:**

(C_5_H_6_N)[ZnCl_3_(C_5_H_5_N)]	*F*(000) = 664
*M**_r_* = 330.97	*D*_x_ = 1.590 Mg m^−^^3^
Monoclinic, *P*2_1_/*n*	Cu *K*α radiation, λ = 1.54184 Å
*a* = 7.80795 (16) Å	Cell parameters from 5372 reflections
*b* = 21.3634 (4) Å	θ = 4.1–67.9°
*c* = 8.90581 (18) Å	µ = 7.60 mm^−^^1^
β = 111.418 (2)°	*T* = 293 K
*V* = 1382.94 (5) Å^3^	Needle, metallic whiteish yellow
*Z* = 4	0.62 × 0.45 × 0.20 mm

### Pyridinium trichlorido(pyridine-κN)zincate(II) Data collection

**Table:**

Rigaku XtaLAB Synergy diffractometer with a HyPix3000 detector	2517 independent reflections
Radiation source: micro-focus sealed X-ray tube, PhotonJet (Cu) X-ray Source	2205 reflections with *I*≥ 2σ(*I*)
Mirror monochromator	*R*_int_ = 0.041
Detector resolution: 10.0000 pixels mm^-1^	θ_max_ = 68.2°, θ_min_ = 4.1°
ω scans	*h* = −9→9
Absorption correction: gaussian (CrysAlis PRO; Rigaku OD, 2023)	*k* = −25→25
*T*_min_ = 0.326, *T*_max_ = 1.000	*l* = −10→10
7075 measured reflections	

### Pyridinium trichlorido(pyridine-κN)zincate(II) Refinement

**Table:**

Refinement on *F*^2^	22 constraints
Least-squares matrix: full	Primary atom site location: dual
*R*[*F*^2^ > 2σ(*F*^2^)] = 0.044	H-atom parameters constrained
*wR*(*F*^2^) = 0.126	*w* = 1/[σ^2^(*F*_o_^2^) + (0.0897*P*)^2^ + 0.0322*P*] where *P* = (*F*_o_^2^ + 2*F*_c_^2^)/3
*S* = 1.02	(Δ/σ)_max_ = 0.0004
2517 reflections	Δρ_max_ = 0.52 e Å^−^^3^
146 parameters	Δρ_min_ = −0.73 e Å^−^^3^
0 restraints	

### Pyridinium trichlorido(pyridine-κN)zincate(II) Fractional atomic coordinates and isotropic or equivalent isotropic displacement parameters (Å^2^)

**Table:**

	*x*	*y*	*z*	*U*_iso_*/*U*_eq_	
Zn1	0.78643 (5)	0.613782 (17)	0.66895 (4)	0.0433 (2)	
Cl1	0.47863 (10)	0.60198 (4)	0.58472 (9)	0.0571 (3)	
Cl2	0.89575 (11)	0.67442 (5)	0.88712 (9)	0.0681 (3)	
Cl3	0.91938 (12)	0.51914 (4)	0.69616 (13)	0.0725 (3)	
N1	0.8423 (3)	0.65916 (11)	0.4893 (2)	0.0421 (5)	
N2	0.5590 (6)	0.46271 (15)	0.7804 (3)	0.0747 (9)	
Hn6	0.5897 (6)	0.48603 (15)	0.7156 (3)	0.0896 (11)*	
C5	0.9977 (4)	0.64895 (15)	0.4623 (3)	0.0506 (7)	
H5	1.0755 (4)	0.61691 (15)	0.5183 (3)	0.0607 (8)*	
C1	0.7294 (5)	0.70374 (14)	0.4048 (4)	0.0554 (8)	
H1	0.6201 (5)	0.71076 (14)	0.4213 (4)	0.0665 (9)*	
C4	1.0465 (5)	0.68401 (18)	0.3550 (4)	0.0632 (9)	
H4	1.1572 (5)	0.67655 (18)	0.3411 (4)	0.0758 (10)*	
C10	0.6846 (6)	0.42697 (19)	0.8822 (6)	0.0783 (11)	
H10	0.8048 (6)	0.42715 (19)	0.8848 (6)	0.0939 (13)*	
C7	0.3359 (5)	0.42816 (17)	0.8751 (4)	0.0644 (9)	
H7	0.2158 (5)	0.42908 (17)	0.8726 (4)	0.0773 (10)*	
C2	0.7691 (6)	0.73986 (17)	0.2938 (4)	0.0696 (10)	
H2	0.6874 (6)	0.77067 (17)	0.2361 (4)	0.0835 (12)*	
C3	0.9304 (6)	0.73001 (17)	0.2689 (4)	0.0697 (10)	
H3	0.9601 (6)	0.75414 (17)	0.1949 (4)	0.0836 (12)*	
C8	0.4629 (5)	0.39052 (18)	0.9809 (5)	0.0695 (10)	
H8	0.4309 (5)	0.36526 (18)	1.0516 (5)	0.0834 (12)*	
C6	0.3854 (6)	0.46435 (17)	0.7731 (4)	0.0726 (10)	
H6	0.2995 (6)	0.49007 (17)	0.6989 (4)	0.0872 (12)*	
C9	0.6370 (6)	0.3901 (2)	0.9827 (6)	0.0858 (13)	
H9	0.7245 (6)	0.3640 (2)	1.0543 (6)	0.1029 (16)*	

### Pyridinium trichlorido(pyridine-κN)zincate(II) Atomic displacement parameters (Å^2^)

**Table:**

	*U*^11^	*U*^22^	*U*^33^	*U*^12^	*U*^13^	*U*^23^
Zn1	0.0393 (3)	0.0496 (3)	0.0467 (3)	−0.00122 (14)	0.0226 (2)	0.00252 (15)
Cl1	0.0372 (4)	0.0717 (5)	0.0634 (5)	−0.0022 (3)	0.0195 (3)	0.0042 (4)
Cl2	0.0604 (5)	0.0938 (7)	0.0588 (4)	−0.0221 (4)	0.0322 (4)	−0.0225 (4)
Cl3	0.0638 (5)	0.0561 (5)	0.1125 (7)	0.0155 (4)	0.0499 (5)	0.0212 (5)
N1	0.0444 (12)	0.0451 (13)	0.0434 (11)	0.0006 (9)	0.0238 (10)	−0.0007 (10)
N2	0.113 (3)	0.0635 (19)	0.0644 (16)	−0.0232 (17)	0.0528 (18)	−0.0023 (14)
C5	0.0450 (16)	0.0613 (19)	0.0499 (14)	0.0019 (13)	0.0226 (13)	0.0012 (13)
C1	0.0649 (19)	0.0510 (18)	0.0584 (16)	0.0149 (14)	0.0320 (15)	0.0062 (14)
C4	0.062 (2)	0.081 (2)	0.0607 (17)	−0.0117 (17)	0.0399 (16)	−0.0027 (18)
C10	0.066 (2)	0.078 (3)	0.112 (3)	−0.0001 (19)	0.057 (2)	0.005 (2)
C7	0.0515 (18)	0.066 (2)	0.081 (2)	−0.0058 (16)	0.0311 (17)	−0.0142 (18)
C2	0.099 (3)	0.057 (2)	0.0588 (18)	0.0164 (18)	0.035 (2)	0.0149 (16)
C3	0.108 (3)	0.060 (2)	0.0545 (16)	−0.019 (2)	0.0451 (19)	−0.0015 (16)
C8	0.071 (2)	0.082 (3)	0.069 (2)	−0.0076 (18)	0.0407 (19)	0.0125 (18)
C6	0.077 (3)	0.059 (2)	0.0627 (19)	−0.0010 (18)	0.0027 (18)	0.0042 (17)
C9	0.062 (2)	0.094 (3)	0.099 (3)	0.0122 (19)	0.026 (2)	0.034 (2)

### Pyridinium trichlorido(pyridine-κN)zincate(II) Geometric parameters (Å, º)

**Table:**

Zn1—Cl1	2.2551 (7)	C4—C3	1.367 (5)
Zn1—Cl2	2.2295 (8)	C10—H10	0.9300
Zn1—Cl3	2.2451 (9)	C10—C9	1.343 (6)
Zn1—N1	2.049 (2)	C7—H7	0.9300
N1—C5	1.338 (3)	C7—C8	1.355 (5)
N1—C1	1.328 (4)	C7—C6	1.352 (5)
N2—Hn6	0.8600	C2—H2	0.9300
N2—C10	1.311 (5)	C2—C3	1.372 (6)
N2—C6	1.333 (6)	C3—H3	0.9300
C5—H5	0.9300	C8—H8	0.9300
C5—C4	1.373 (4)	C8—C9	1.353 (6)
C1—H1	0.9300	C6—H6	0.9300
C1—C2	1.376 (4)	C9—H9	0.9300
C4—H4	0.9300		
			
*Cg*1···*Cg*2^i^	4.051 (2)	*Cg*2···*Cg*2^ii^	3.824 (2)
			
Cl2—Zn1—Cl1	112.16 (3)	H10—C10—N2	120.5 (2)
Cl3—Zn1—Cl1	109.25 (3)	C9—C10—N2	118.9 (4)
Cl3—Zn1—Cl2	114.48 (4)	C9—C10—H10	120.5 (3)
N1—Zn1—Cl1	107.33 (6)	C8—C7—H7	120.3 (2)
N1—Zn1—Cl2	106.42 (7)	C6—C7—H7	120.3 (2)
N1—Zn1—Cl3	106.76 (7)	C6—C7—C8	119.4 (4)
C5—N1—Zn1	121.9 (2)	H2—C2—C1	120.3 (2)
C1—N1—Zn1	119.67 (18)	C3—C2—C1	119.4 (3)
C1—N1—C5	118.2 (2)	C3—C2—H2	120.3 (2)
C10—N2—Hn6	118.7 (2)	C2—C3—C4	118.7 (3)
C6—N2—Hn6	118.7 (2)	H3—C3—C4	120.67 (19)
C6—N2—C10	122.6 (3)	H3—C3—C2	120.7 (2)
H5—C5—N1	118.77 (17)	H8—C8—C7	120.4 (2)
C4—C5—N1	122.5 (3)	C9—C8—C7	119.3 (4)
C4—C5—H5	118.8 (2)	C9—C8—H8	120.4 (2)
H1—C1—N1	118.92 (16)	C7—C6—N2	119.2 (3)
C2—C1—N1	122.2 (3)	H6—C6—N2	120.4 (2)
C2—C1—H1	118.9 (2)	H6—C6—C7	120.4 (2)
H4—C4—C5	120.4 (2)	C8—C9—C10	120.6 (4)
C3—C4—C5	119.1 (3)	H9—C9—C10	119.7 (3)
C3—C4—H4	120.44 (19)	H9—C9—C8	119.7 (2)
			
Zn1—N1—C5—C4	−172.4 (2)	C5—C4—C3—C2	0.5 (4)
Zn1—N1—C1—C2	173.6 (2)	C1—N1—C5—C4	2.1 (3)
N1—C5—C4—C3	−1.8 (4)	C1—C2—C3—C4	0.5 (4)
N1—C1—C2—C3	−0.2 (4)	C10—N2—C6—C7	0.4 (4)
N2—C10—C9—C8	−1.1 (5)	C10—C9—C8—C7	0.7 (6)
N2—C6—C7—C8	−0.8 (4)	C6—N2—C10—C9	0.5 (4)
C5—N1—C1—C2	−1.1 (3)	C6—C7—C8—C9	0.2 (4)

Symmetry codes: (i) −*x*+1, −*y*+1, −*z*+1; (ii) −*x*+1, −*y*+1, −*z*+2.

### Pyridinium trichlorido(pyridine-κN)zincate(II) Hydrogen-bond geometry (Å, º)

**Table:**

*D*—H···*A*	*D*—H	H···*A*	*D*···*A*	*D*—H···*A*
N2—HN6···Cl1	0.86 (1)	2.74 (1)	3.389 (3)	133 (1)
N2—HN6···Cl3	0.86 (1)	2.74 (1)	3.388 (5)	134 (1)
C8—H8···Cl2^ii^	0.93 (1)	2.92 (1)	3.681 (5)	140 (1)
C9—H9···Cl2^iii^	0.93 (1)	2.93 (1)	3.669 (5)	137 (1)

Symmetry codes: (ii) −*x*+1, −*y*+1, −*z*+2; (iii) −*x*+2, −*y*+1, −*z*+2.
